# *Paraburkholderia sabiae* Uses One Type VI Secretion System (T6SS-1) as a Powerful Weapon against Notorious Plant Pathogens

**DOI:** 10.1128/spectrum.01622-23

**Published:** 2023-07-13

**Authors:** Sebastian Hug, Benjamin Heiniger, Kim Bolli, Sarah Paszti, Leo Eberl, Christian H. Ahrens, Gabriella Pessi

**Affiliations:** a Department of Plant and Microbial Biology, University of Zürich, Zurich, Switzerland; b Agroscope – Molecular Ecology, Swiss Institute of Bioinformatics, Zurich, Switzerland; Universita degli Studi Roma Tre Dipartimento di Scienze

**Keywords:** rhizobium, competition, phytopathogen, killing, biocontrol, potato, complete genome

## Abstract

Paraburkholderia sabiae LMG24235 is a nitrogen-fixing betaproteobacterium originally isolated from a root nodule of Mimosa caesalpiniifolia in Brazil. We show here that this strain effectively kills strains from several bacterial families (*Burkholderiaceae*, *Pseudomonadaceae*, *Enterobacteriaceae*) which include important plant pathogens in a contact-dependent manner. *De novo* assembly of the first complete genome of *P. sabiae* using long sequencing reads and subsequent annotation revealed two gene clusters predicted to encode type VI secretion systems (T6SS), which we named T6SS-1 and T6SS-3 according to previous classification methods (G. Shalom, J. G. Shaw, and M. S. Thomas, Microbiology, 153:2689–2699, 2007, https://doi.org/10.1099/mic.0.2007/006585-0). We created *P. sabiae* with mutations in each of the two T6SS gene clusters that abrogated their function, and the T6SS-1 mutant was no longer able to outcompete other strains in a contact-dependent manner. Notably, our analysis revealed that T6SS-1 is essential for competition against several important plant pathogens *in vitro,* including Burkholderia plantarii, Ralstonia solanacearum, Pseudomonas syringae, and Pectobacterium carotovorum. The 9-log reduction in P. syringae cells in the presence of *P. sabiae* was particularly remarkable. Importantly, in an *in vivo* assay, *P. sabiae* was able to protect potato tubers from bacterial soft rot disease caused by *P. carotovorum*, and this protection was partly dependent on T6SS-1.

**IMPORTANCE** Rhizobia often display additional beneficial traits such as the production of plant hormones and the acquisition of limited essential nutrients that improve plant growth and enhance plant yields. Here, we show that the rhizobial strain *P. sabiae* antagonizes important phytopathogens such as *P. carotovorum,* P. syringae, and R. solanacearum and that this effect is due to contact-dependent killing mediated by one of two T6SS systems identified in the complete, *de novo* assembled genome sequence of *P. sabiae*. Importantly, co-inoculation of Solanum tuberosum tubers with *P. sabiae* also resulted in a drastic reduction of soft rot caused by *P. carotovorum* in an *in vivo* model system. This result highlights the protective potential of *P. sabiae* against important bacterial plant diseases, which makes it a valuable candidate for application as a biocontrol agent. It also emphasizes the particular potential of rhizobial inoculants that combine several beneficial effects such as plant growth promotion and biocontrol for sustainable agriculture.

## INTRODUCTION

Rhizobia are symbiotic nitrogen-fixing bacteria which associate with legumes and form root or stem nodules where they convert inert atmospheric dinitrogen (N_2_) into biologically available ammonia (NH_3_) (1). Rhizobia are polyphyletic and include alphaproteobacteria (alpha-rhizobia) and betaproteobacteria from the *Burkholderiaceae* family (beta-rhizobia) (2). In 2014, the genus *Burkholderia* sensu lato was divided into two new genera, *Burkholderia* sensu stricto and *Paraburkholderia* (3). The *Burkholderia* sensu stricto clade covers phytopathogens such as Burkholderia gladioli, which causes onion soft rot, and clinically relevant species such as members of the Burkholderia cepacia complex (3, 4). In contrast, the genus *Paraburkholderia* contains environmental plant beneficial strains such as nitrogen-fixing and plant growth-promoting symbionts (e.g., Paraburkholderia phymatum, Paraburkholderia sabiae, Paraburkholderia tuberum, Paraburkholderia tropica, Paraburkholderia phytofirmans, and Paraburkholderia kururiensis) (3, 5–8). Rhizobia must be competitive in order to prevent other strains present in the soil from colonizing plant roots. To be competitive they use various strategies, one of which involves the killing of competitors. Our group previously showed that *P. phymatum*’s high competitiveness against other beta-rhizobial strains was partly dependent on the function of specific type VI secretion systems (T6SSs). In fact, T6SS mutants showed reduced fitness not only in *in vitro* interbacterial competition assays but also in terms of legume root nodule occupancy (9). T6SS clusters typically contain at least 13 genes coding for core components that ensure the functionality of the secretion system (10). The complex is made up of the baseplate (TssAEFGK), membrane complex (TssJLM), sheath (TssBC), tail tube (TssD or Hcp), and tail tip (TssI or VgrG) (11). The tail sheath is recycled after firing by the ATPase TssH (ClpV) (12). Additional *tssI* genes can often be found in genomic regions outside the T6SS clusters, in small clusters containing an additional effector/immunity protein pair (13). The effectors for competition can be differentiated into four classes: membrane-targeting lipases, DNA/RNA-targeting nucleases, cell wall-degrading enzymes (muramidase, amidase), and cytoplasmic targeting molecules (14). The VgrG of the T6SS can additionally be decorated by proteins with attached effector proteins from the proline-alanine-alanine-arginine (PAAR) repeat superfamily (15). This PAAR domain is also often found in Rhs-repeat containing proteins, which completely enclose the active effector domain (15, 16). T6SSs have mainly been studied in pathogens, where they have been shown to have versatile roles in the production of different virulence factors, including motility in Vibrio cholerae (17), biofilm formation in Pseudomonas aeruginosa (18), and dominance in multi-species biofilms (19). They also play a role in the transport of metal ions such as zinc, manganese, iron, copper, and molybdate (20). However, the key function of this secretion system is to deliver toxins into prokaryotic or eukaryotic target cells (21). The T6SS usually depends on cell-cell contact because the attacker must be in proximity to the target for successful injection of effector proteins (22). Recently, however, the first contact-independent T6SS toxic effector was described in Yersinia pseudotuberculosis (23). T6SSs are found in one-fourth of all Gram-negative bacteria and were recently also shown to be able to target and kill Gram-positive bacteria (24).

In this study, we show that the nitrogen-fixing and nodulating strain *P. sabiae* LMG24235 (25) can outcompete strains from several genera, including *Burkholderia*, Pseudomonas, *Pectobacterium*, and *Ralstonia*. Genome sequencing using long-read data from the PacBio platform, *de novo* assembly, annotation and downstream functional genomic analysis identified two T6SS on chromosome 2. Mutant analysis established that T6SS-1 is essential for competition against phytopathogens such as Pectobacterium carotovorum, which belongs to the soft rot *Enterobacteriaceae* (SRE). SRE pathogens cause soft rot in 50% of angiosperms, including the important crop plants potato, tomato, and maize (26). Finally, a co-inoculation assay on potato tubers (Solanum tuberosum) showed that *P. sabiae* effectively protects the tuber from bacterial soft rot caused by *P. carotovorum* and that this protective effect is at least partially dependent on T6SS-1.

## RESULTS

### *P. sabiae* exhibits antagonistic activity against a wide range of bacteria from different genera.

**FIG 1:**
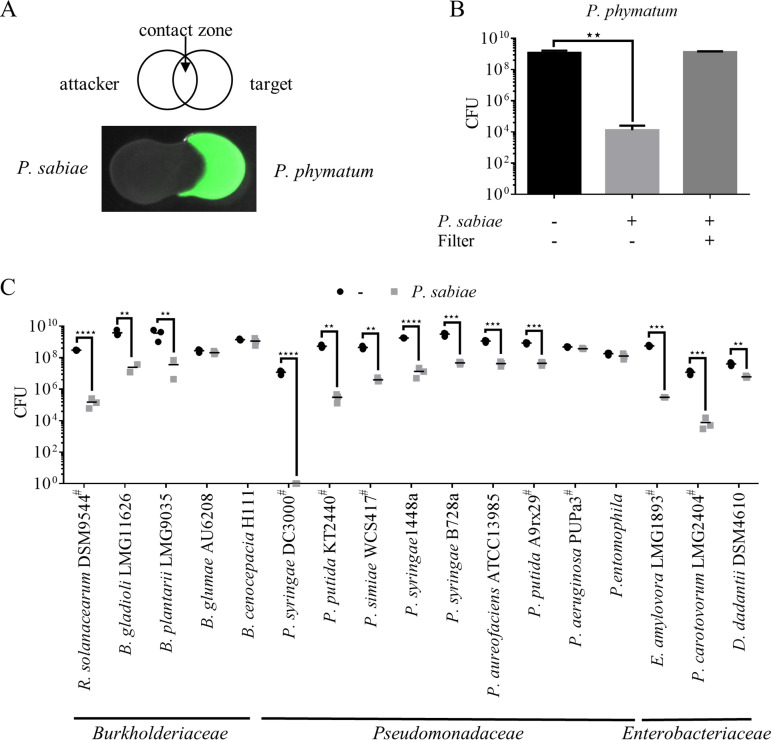
*P. sabiae* outcompetes a wide range of Gram-negative bacteria. (A) Drop assay between *P. sabiae* and *P. phymatum*. The clear contact zone in the middle indicates dead target bacteria (GFP-labeled). (B) Competition assay between *P. sabiae* and *P. phymatum.* The CFU of *P. phymatum* was determined with/without co-inoculation with *P. sabiae* after 24 h incubation at 28°C. Without cell-cell contact, no killing was observed. A one-way ANOVA was performed on the results obtained from 3 biological replicates (*n* = 3, **, *P* < 0.01). (C) A wide range of bacteria belonging to three families were outcompeted by *P. sabiae* to varying degrees. The CFU of the target strain (*y* axis) without (black circles) or in the presence of the attacker *P. sabiae* (gray squares) is shown; data are the means from 3 independent biological replicates. Target strains marked with a hash symbol (#) are chromosomally tagged.

In previous competition experiments using the very competitive strain *P. phymatum* as an attacker and *P. sabiae* as a target, we observed that *P. sabiae* was able to kill *P. phymatum* (9, 27). In fact, after 24 h of co-incubation, less *P. phymatum* cells were observed in the zone where the two cultures overlapped (Fig. 1A). CFU quantification after co-inoculation at a 10:1 (attacker:target) ratio on a full medium plate for 24 h showed a significant 4- to 5-log reduction in *P. phymatum* (target) in the presence of *P. sabiae* (Fig. 1B). In the presence of a 0.2-μm pore size filter between the attacker and the target strain, no killing was detected, suggesting that this antagonistic activity is dependent on cell-cell contact. To test the range of bacteria outcompeted by *P. sabiae*, we performed competition assays with strains from three different families, encompassing phytopathogens and plant symbionts from our laboratory strain collection (Table S1), including *Burkholderiaceae* (28) (*B. gladioli* LMG11626, Burkholderia glumae AU6208, Burkholderia plantarii LMG9035, and Ralstonia solanacearum DSM9544), *Pseudomonadaceae* (Pseudomonas syringae DC3000, Pseudomonas putida KT2440, Pseudomonas simiae WCS417, P. syringae 1448a, P. syringae B728a, Pseudomonas aureofaciens ATCC 12985, P. putida A9rx29, P. aeruginosa PUPa3, and Pseudomonas entomophila) and *Enterobacteriaceae* (Erwinia amylovora LMG1893, P. carotovorum LMG2404, and Dickeya dadantii DSM4610) (Fig. 1C). Antagonized strains were identified in all three families. We focused on the phytopathogens, which include three strains belonging to the *Burkholderiaceae* family. *B. gladioli* and *B. plantarii* were outcompeted by *P. sabiae* with a 2- and 3-log reduction of CFU, respectively. R. solanacearum, the causative agent of bacterial wilt (29), was also found to be affected by the presence of *P. sabiae*, showing a 2-log reduction of CFU after 24 h co-incubation on plate. Among the *Pseudomonadaceae*, P. syringae DC3000 (30), an important pathogen of tomato, was almost completely eliminated in the presence of *P. sabiae*, as evidenced by a 9-log CFU decrease compared to P. syringae cells incubated without *P. sabiae*. Two out of three tested *Enterobacteriaceae*, *P. carotovorum* LMG2404, a soft root-causing phytopathogen (31), and E. amylovora, the causative agent of fire blight (32), showed a 4-log decrease in CFU in the presence of *P. sabiae*. In contrast, the effect of *P. sabiae* on the soft root pathogen *D. dadantii* was only marginal, with less than a 1-log CFU reduction after co-inoculation. We also identified several strains resistant to *P. sabiae* attack, such as the rice pathogen B. glumae AU6208 (33), the opportunistic pathogen Burkholderia cenocepacia H111, and two Pseudomonas strains with biocontrol properties (P. aeruginosa PUPa3 and *P. entomophila*) (34, 35).

### Genome sequencing, *de novo* assembly, and analysis for secretion systems.

**FIG 2:**
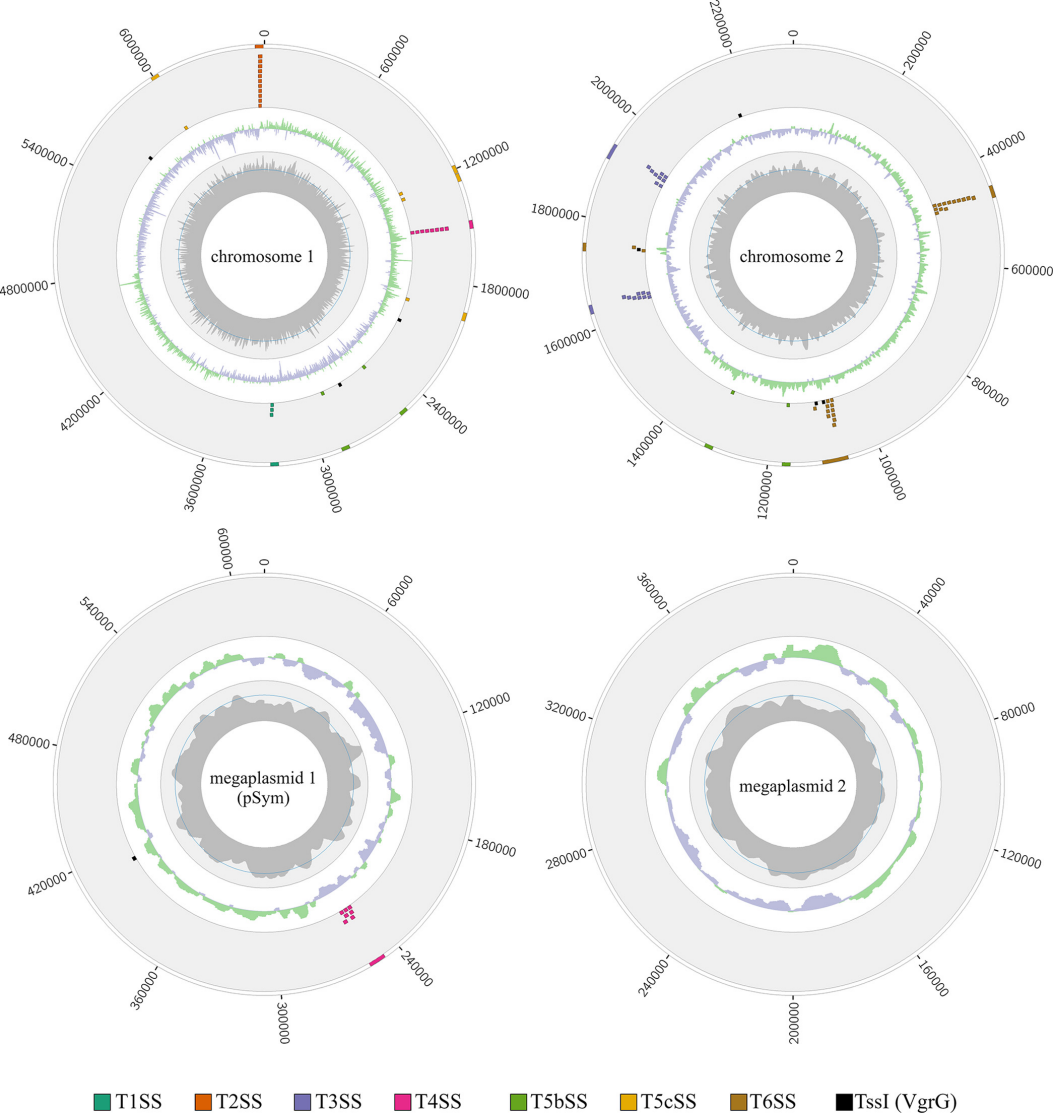
Circos plots of the four replicons (two chromosomes and two megaplasmids) of the *P. sabiae* LMG24235 genome showing the genomic location of predicted secretion systems and their corresponding genes (two outer circles, color legend at the bottom). The two inner circles show the GC skew (green for positive and violet for negative) and PacBio read coverage including the mean (dark blue line).

To unravel the mechanism(s) that could account for the observed contact-dependent killing activity, the genome of *P. sabiae* LMG24235 was sequenced and *de novo* assembled using 3rd generation long reads from the PacBio platform (see Table 1 for an overview of selected genome features). The genome consists of 4 replicons with a total size of 9.9 MB; chromosome 1 (6,584,006 bp, 5,913 genes, 62.5% GC), chromosome 2 (2,309,677 bp, 2,001 genes, 62.2% GC), megaplasmid 1 (pSym; 615,774, 553 genes, 58.4% GC) and megaplasmid 2 (399,867 bp, 392 genes, 59.5% GC) (Fig. 2).

**TABLE 1:** Selected characteristics of the four replicons of the *P. sabiae* LMG24235 genome^*a*^

Replicon		%GC	Genes	CDS	Pseudo-genes	rRNA	tRNA	ncRNA	tmRNA
Name	Length (bp)								
Chromosome 1	6,584,006	62.51	5,913	5,828	126	18	63	3	1
Chromosome 2	2,309,677	62.17	2,001	2,000	62		1		
Megaplasmid 1	615,744	58.44	553	553	74				
Megaplasmid 2	399,867	59.51	392	392	34				
Total	9,909,294	62.06	8,859	8,773	296	18	64	3	1

a ncRNA, non-coding RNA; tmRNA, transfer-messenger RNA.

**FIG 3:**
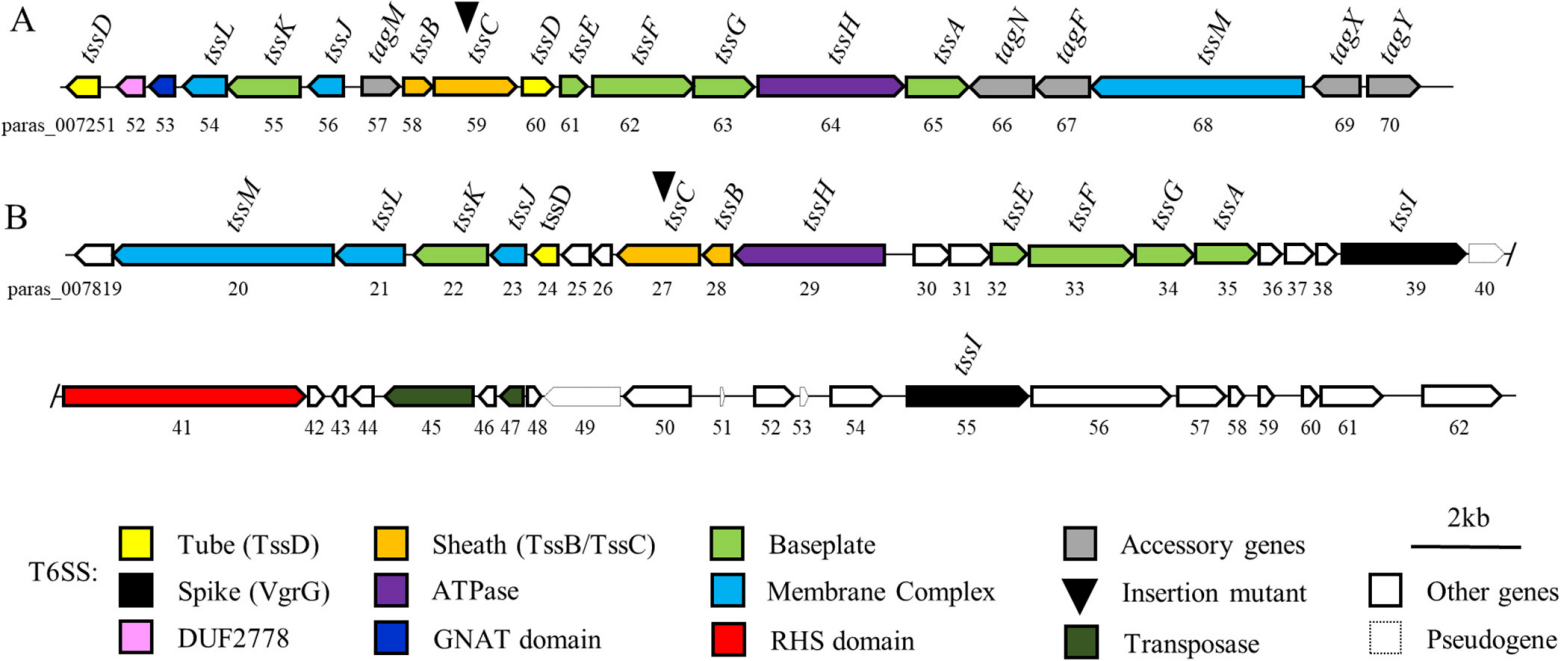
Physical map of the two T6SS loci of *P. sabiae* (A) T6SS-1 and (B) T6SS-3. The mutated *tssC* gene in each cluster is labeled by a triangle (▾). The nomenclature of the accessory genes was taken from Spiewak et al. (45).

The megaplasmid 1 seems to contain all genes necessary to establish a functional nitrogen-fixing symbiosis with legumes (Table S2). Several secretion systems (T1SS to T6SS) were predicted in the genome using TXSScan (36). They are listed in Table S3 and are also shown in Fig. 2, together with the *tssI* genes and T6SS VgrG effector genes. We focused on identifying and analyzing the T6SS using SeCreT6 (https://bioinfo-mml.sjtu.edu.cn/SecReT6/index.php) (37) because our data showed that the competition is dependent on cell-cell contact. Two T6SS gene clusters were identified on chromosome 2 which, based on their synteny to established T6SS types (38, 39), were named T6SS-1 (*paras_007251* to *paras_007268*) and T6SS-3 (*paras_007819* to *paras_007842*) (Fig. 3). T6SS-1 contains all the accessory genes (*tagMNXY*) considered hallmarks for T6SS-1 (39). Both clusters encode 12 of the 13 core genes required to form a functional T6SS, including genes for the baseplate, membrane complex, and tube. The genes downstream of *tssL* in the T6SS-1 cluster (*paras_007252* and *paras_007253*) code for proteins with a domain of unknown function (DUF) called DUF2778 and a GNAT domain, respectively. However, only the T6SS-3 cluster (Fig. 3) contains two genes encoding the tail tip protein VgrG (*tssI*, *paras_007839*, and *paras_007855*). The *tssI* gene can often be found outside the T6SS clusters, normally together with an additional effector/immunity protein pair (13). In addition to the two *tssI* genes in the T6SS-3 cluster, the PGAP annotation revealed the presence of six additional *tssI* copies distributed throughout the *P. sabiae* genome (*paras_000364*, *paras_002442*, *paras_002998*, *paras_005637*, *paras_008381*, and *paras_008754*) (Fig. 2). These *tssI* are always followed by a gene coding for an effector (Fig. S1). Three *tssI*, *paras_000364*, *paras_002998* and *paras_008380*, are followed by *tle*1, which encodes a putative membrane targeting phospholipase (40). The cluster containing the *tssI paras_005637* encodes a potential DNA-targeting VRR-NUC nuclease as well as the respective immunity protein (TsiV; DUF3396), (41) and the cluster containing the *tssI paras_008754* harbors a gene coding for an effector with a cell wall-targeting peptidoglycan hydrolase. The last *tssI* (*paras_002442*) cluster encodes an RHS domain-containing protein with an unknown target. The T6SS-1 and T6SS-3 clusters were classified by TXSScan as T6SS-families i4b and i3, respectively (37). While the T6SS-3 cluster is organized in the form of two potential operons (*tssMLKJDCBH* and *tssEFGAI*) (Fig. 3A), the T6SS-1 consists of three potential operons (*tssLKJ*, *tssBCDEFGHA*, and *tssM*) (Fig. 3B).

### *P. sabiae* competitiveness is dependent on T6SS-1.

**FIG 4:**
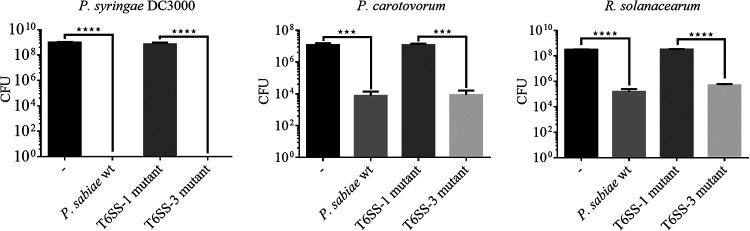
*P. sabiae* uses T6SS-1 to antagonize other strains. Interbacterial competition with *P. sabiae* as attacker (wild-type, T6SS-1 mutant and T6SS-3 mutant) and three target strains (the phytopathogens P. syringae DC3000, *P. carotovorum*, and R. solanacearum). The CFU of the respective target strains are shown (3 biological replicates). An ANOVA with Tukey’s multiple-comparison test was performed to assess the statistical significance of the observed results (***, *P* ≤ 0.001; ****, *P* ≤ 0.0001). Target strains are chromosomally tagged with *gfp*.

To determine whether one or both T6SS was responsible for outcompeting the other strains by contact-dependent killing, we created insertion mutants in the *tssC* gene of the two T6SS clusters (T6SS-1: *paras_007259* and T6SS-3: *paras_007827*). The TssC protein product is the large subunit of the sheath, which is essential for contraction, leading to the ejection of the tube (42). After confirming that the mutants behaved similarly in terms of growth (Fig. S2), *P. sabiae* wild-type and mutant strains (called T6SS-1_IM and T6SS-3_IM) were tested in competition assays with the three important phytopathogens P. syringae DC3000, *P. carotovorum* LMG2404, and R. solanacearum DSM9544 as targets (Fig. 4). The T6SS-1 mutant (*tssC*, *paras_007259*) completely lost the ability to outcompete the three phytopathogens and displayed similar CFU counts as the strain incubated in the absence of the attacker strain *P. sabiae*. The T6SS-1 dependency of *P. sabiae*’s inhibitory effect could also be shown for all other susceptible target strains of the *Pseudomonadaceae*, *Enterobacteriaceae*, and *Burkholderiaceae* families (Table S4). In contrast, the T6SS-3 mutant was as competitive as the wild type, suggesting that T6SS-3 was not important for *P. sabiae*’s killing ability of the tested strains.

### Expression of the T6SS-1 gene cluster.

**FIG 5:**
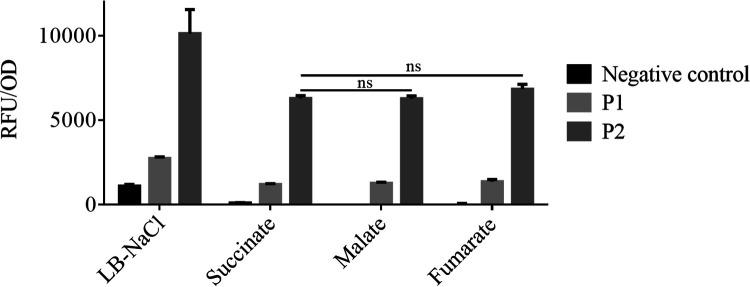
Quantification of *P. sabiae* T6SS-1 expression using a GFP reporter. The GFP expression of the T6SS-1 promoter fusions P1 (expression of *tssLKJ*) and P2 (expression of *tssBCDEFGHA*) was measured with a plate reader (Tecan Infinite M200 Pro) after 48 h at 28°C. Expression was normalized to the OD_600_. A two-way ANOVA with Tukey’s multiple-comparison test was used; no significant difference in expression was found for P1 and P2 constructs when cells were grown in minimal medium containing either succinate, malate, or fumarate.

To gain insight into the expression of the T6SS-1 cluster, we fused the promoter region for the two larger, putative operons of T6SS-1 (P1: *tssLKJ*, *paras_007251-paras_007256* and P2: *tssBCDEFGHA*, *paras_007257-paras_007265*) to the gene coding for a green fluorescent protein (GFP). The reporter strains were grown in complex and minimal media with different C4-dicarboxylates as carbon sources (succinate, malate, fumarate) at 28°C for 48 h. We chose C4-dicarboxylates because they are the major carbon and energy source given to rhizobia by the plant during symbiosis. In all media tested, the activity of the P2 promoter driving expression of the genes coding for TssB, TssC, TssD, TssE, TssF, TssG, TssH, and TassA was about 3-fold higher than that of P1 promoter transcribing the other operon of the cluster (Fig. 5). For both P1 and P2 constructs, the maximal expression was observed in complex medium (lysogeny broth [LB] without salt) and the identity of the added C4 carbon source did not influence the expression levels.

### Conservation analysis of T6SS-1.

**FIG 6:**
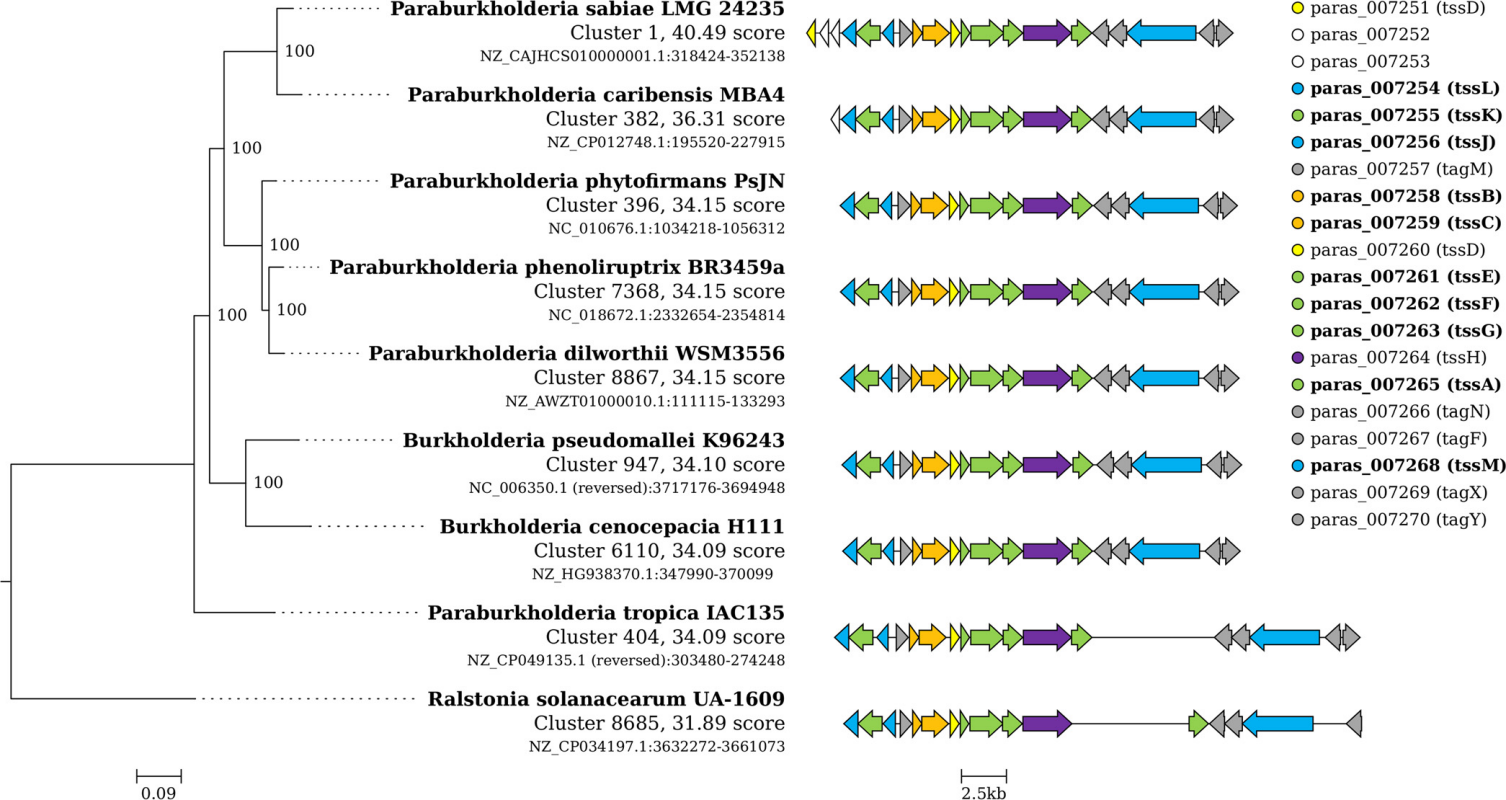
A phylogenetic tree and synteny plot from eight strains of interest. A cBlaster analysis was performed with 10 of the T6SS core genes and the 9 highest-scoring strains (6 from the genus *Paraburkholderia*, 2 from *Burkholderia*, and Ralstonia solanacearum) are shown along with the gene cluster identifier, cBlaster score, accession number, and genomic coordinates of the gene clusters identified. The legend shows 21 genes, including the 10 query genes (shown in bold).

We next explored the most similar gene clusters to the *P. sabiae* T6SS-1 using cBlaster as described previously (43). For this, we required 10 of the 13 core genes to be present within 20 kb (the two genes making up the tail were excluded because the tail [*tssD*] and tip [*tssI*] can be located outside the operon, as well as *tssH*). T6SS-1 was identified in nearly all *Burkholderia* species and in many *Paraburkholderia* species. Next, we extended the T6SS-1 *in silico* analysis to include all bacteria. All the T6SS-1 clusters were found in certain orders of the class *Gammaproteobacteria* (*Oceanospirrales*, *Pseudomonadales*, *Salinisphaerales*, and *Xanthomonadales*) and *Betaproteobacteria* (*Burkholderiales*, *Neisseriales*, and *Rhodocyclales*). Out of the 4,869 identified T6SS-1 clusters, 97.4% were found in *Burkholderiales*. The three genera *Burkholderia*, *Paraburkholderia*, and *Caballeronia* showed different frequencies of *vgrG* containing T6SS-1. While most *Burkholderia* (95.9%) contain a *vgrG* in the cluster, *vgrG* was only found in 50% of *Caballeronia* and 28.8% of *Paraburkholderia* strains (e.g., *P. tropica* IAC5). A phylogenetic tree and a synteny plot of the identified clusters from eight strains of interest were generated (Fig. 6). These eight strains include human pathogens (Burkholderia pseudomallei, B. cenocepacia H111), phytopathogens (Ralstonia solanacearum), and plant growth-promoting strains (e.g., Paraburkholderia phenoliruptrix, Paraburkholderia dilworthii, and Paraburkholderia phytofirmans).

### Biocontrol activity of *P. sabiae* against *P. carotovorum* in a potato tuber infection model.

**FIG 7:**
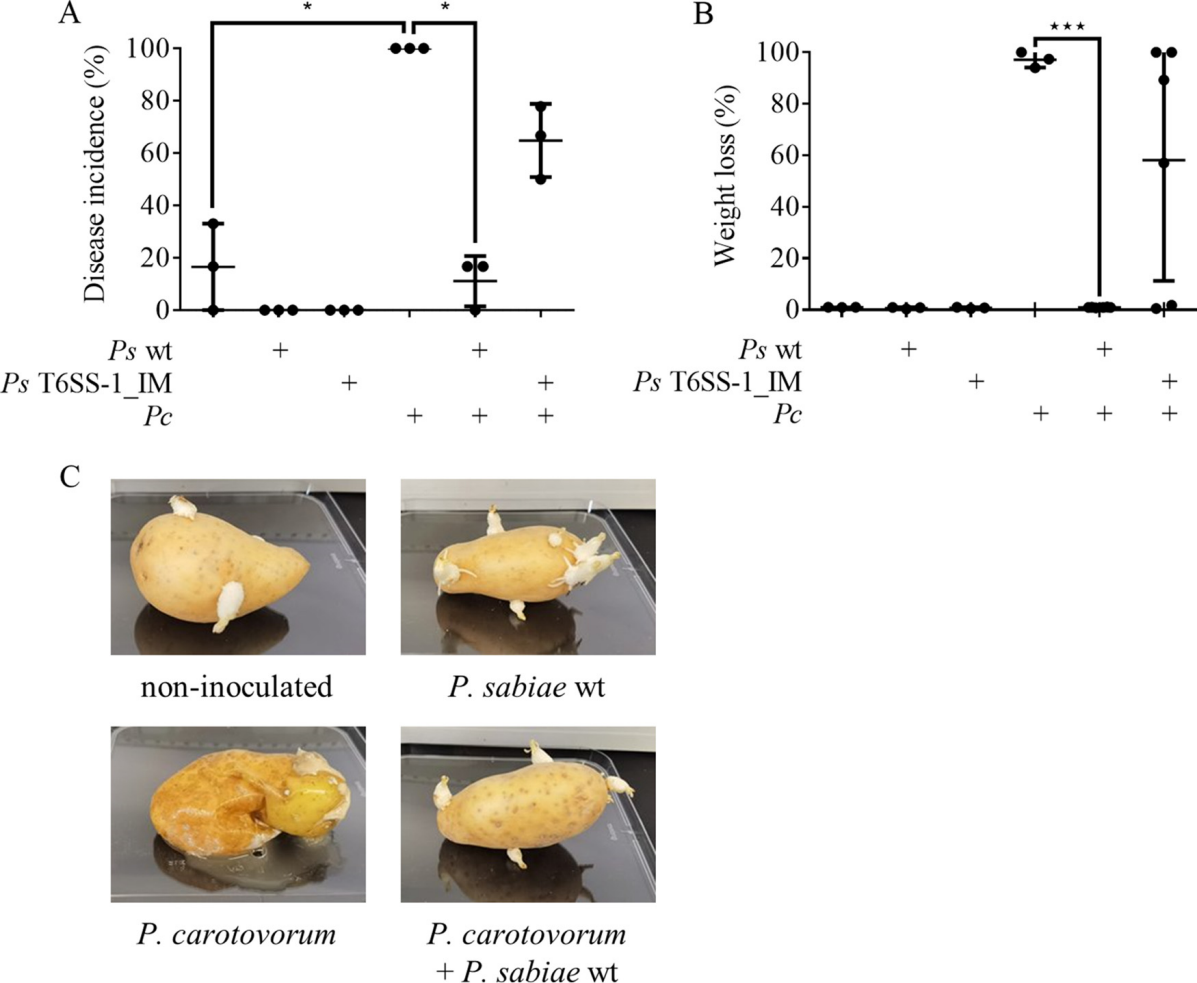
*P. sabiae* protects potato tubers against the phytopathogen *P. carotovorum*. Potato tubers were inoculated and incubated at 28°C for 2 weeks. The co-inoculation was performed at the same spot. First, the biocontrol strain *P. sabiae* wild type (*Ps* wt) or *P. sabiae* T6SS-1 mutant (*Ps* T6SS-1_IM) was injected with a pipette tip to inflict mechanical damage to the potato. After 30 min, the soft rot bacterium *P. carotovorum* (*Pc*) was added to the mechanically damaged spot. The disease incidence of the potato tubers of (A) cv. Celtiane is shown. (B) Rotten tissue was removed from the potato tubers (Celtiane) and the healthy tissue was weighed. A one-way ANOVA with Tukey’s multiple-comparison test was used to analyze biological triplicates (controls, 1 potato/replicate; competition, 2 potatoes/replicate). (C) A representative replicate of the injected potato tubers (Celtiane). An ANOVA with Tukey’s multiple-comparison test was performed (***, *P* ≤ 0.001; ****, *P* ≤ 0.0001).

To further investigate the competitiveness of *P*. *sabiae*, we conducted competition experiments in a more natural environment of *P. carotovorum*, a pathogen that causes soft root in several important crops. To this end, we used potato tubers (cv. Celtiane) as model system because they are easy to handle and of agricultural relevance. We first injected the attacker strain *P. sabiae* (10^6^ cells) via a pipette tip into the potato tuber; after 30 min, we added the target strain *P. carotovorum* (10^5^ cells) on the same spot. The potatoes were then incubated at 28°C for 2 weeks before disease incidence was determined (Fig. 7A). For the potato tubers, disease severity was determined by removing the rotten soft tissue with a scalpel and weighing the remaining firm tissue (Fig. 7B, Fig. S3). While *P. sabiae* was not harmful to the potato tuber and survived on the tubers for at least 2 weeks, reaching 10^7^ CFU at the infection site (Fig. S4), *P. carotovorum* completely macerated the tubers. The co-inoculation of the pathogen with wild-type *P. sabiae* was able to significantly lower the disease incidence in the potato tubers (Celtiane) to a range of 0% to 17% (Fig. 7A). Another cultivar of potato tubers (Anabelle) was tested and showed a reduction in disease incidence (Fig. S5). To evaluate a possible contribution of the T6SS-1 to the protection of the tuber against phytopathogen attack, the tubers were co-inoculated with *P. carotovorum* and the *P. sabiae* T6SS-1 mutant. *P. sabiae* T6SS-1_IM showed partial protection, decreasing the disease incidence to roughly 67% in Celtiane as well as in Anabelle (Fig. 7B and Fig. S5). This result suggests that T6SS-1 is at least partially responsible for the protective effect of *P. sabiae* against potato soft rot caused by *P. carotovorum*.

## DISCUSSION

In this study, we report the first complete genome of *P. sabiae*, a *Paraburkholderia* strain described in 2008 as a nitrogen-fixing symbiont from “sabià”, the Portuguese name of the mimosa tree. A complete genome represents the optimal basis to identify the full complement of genes: a recent study illustrated this advantage for P. aeruginosa MPAO1, where a fragmented Illumina short read-based assembly covered 99.3% of the complete PacBio-based genome sequence, but missed 3 of the 10 VgrG genes, important T6SS effectors, as well as other important genes encoding NRPS (44). We show here that this beta-rhizobial strain kills a wide range of bacteria in a contact-dependent manner. Genome mining allowed us to identify two T6SS, which were subjected to mutagenesis. Indeed, one of *P. sabiae*’s T6SS was revealed to be responsible for the killing and was named T6SS-1, since it is very similar to the T6SS-1 of B. pseudomallei and Burkholderia thailandensis as well as to the T6SS present in most B. cenocepacia strains (39, 45). T6SS-1 in these strains was shown to contribute to pathogenesis (46) and to play a role in bacterial competition (38). Moreover, T6SS-1 is present in several *Paraburkholderia* species, including *P. dilworthii*, *P. phenoliruptrix*, and the biocontrol strain *P. phytofirmans* (Fig. 6). T6SS-1 was reported to be located on the chromosomes of most *Paraburkholderia* (38), but we found multiple Paraburkholderia caribensis species (e.g., MBA4, DSM13236), which harbor the cluster on their plasmids. The T6SS-1 cluster of *P. sabiae*, as well as most of the orthologous clusters in *Paraburkholderia* strains, do not include a *vgrG* (*tssI*) gene. Nevertheless, a gene coding for a potential effector with a DUF2778 domain (*paras007252*) was found downstream of *tssL*. This DUF2778 domain is present on the Tlde1 effector of Salmonella typhimurium. Tlde1 targets the peptidoglycan layer and has carboxypeptidase and transpeptidase activity, leading to altered cell division, swelling, and lysis (40). The gene coding for the immunity protein (Tldi1) is located upstream of the effector gene in *S. typhimurium*, but was not found in *P. sabiae*. Only strains belonging to the *Burkholderiaceae* family (*Burkholderia* [95.9%], *Caballeronia* [50%], and *Paraburkholderia* [28,8%]) contain *vgrG* in the T6SS-1 cluster (e.g., P. tropica IAC135, Fig. 6). A closer look into the T6SS-1 cluster showed the presence of a gene (*paras_007257*) upstream of *tssBC*, coding for a protein with a tetratrico peptide repeat (TPR) motif that is reported to promote protein interactions and is found in proteins with various functions (47). Roughly 80% of the i4b subtype T6SS clusters (37) encode a TPR containing periplasmatic Type six secretion dynamic localization protein A (TslA), which has been shown to be required for proper localization of the T6SS (48). TPR motifs are also found in T6SS immunity proteins such as SelE in Klebsiella pneumoniae (49). However, the identity of the immunity protein(s) that *P. sabiae* uses to protect itself and which effector(s) *P. sabiae*’s T6SS-1 uses to kill different phytopathogens are still unknown. Mutagenesis of each of the six additional *vgrG* clusters in the genome (*paras_000364*, *paras_002442*, *paras_002998*, *paras_005637*, *paras_008381*, and *paras_008754*) would be the next step to reveal the identity of the effector(s) that cause the antagonistic effects. Interestingly, P. putida KT2440, which has shown T6SS-mediated protective effects against various phytopathogens such as Xanthomonas campestris or *P. carotovorum* (50), encodes two gene clusters coding for effectors similar to *paras_002445* and *paras_007841* (PP_RS16160: 30.95% identity; PP_RS21210: 27.8% identity). *paras_002445* and *paras_007841* code for proteins of unknown function containing a Rearrangement hot spot (Rhs) and a DUF6351 domain. Moreover, several other strains that were sensitive to *P. sabiae* attack have been shown to encode and use a T6SS to compete with other bacteria (50, 51). For example, P. syringae pv. tomato DC3000 encodes two T6SS, HS-I and HS-II, and HS-I has been shown to be important for competition against other plant-associated bacteria such as Agrobacterium tumefaciens and *D. dadantii* (51). Whether T6SS-positive strains respond to *P. sabiae* attack by firing back (“tit for tat” response [52]) is an interesting question that remains to be answered. Notably, B. cenocepacia H111, which possesses a similar T6SS-1 (38), is not affected in terms of growth after co-inoculation with *P. sabiae* (Fig. 1). However, more research is needed to investigate whether B. cenocepacia H111 expresses an immunity protein that protects this strain from attack by *P. sabiae* or whether the strain kills *P. sabiae*.

The phenotypic characterization of the T6SS-1 mutant strain did not show any significant differences in biofilm formation, motility, resistance to salt stress, and symbiotic properties (using Mimosa pudica as the host plant), suggesting that *P. sabiae* T6SS-1 is not involved in regulating traits other than interbacterial competition (Table S5). Since T6SS-1 is involved in pathogenicity in several *Burkholderia* strains, the pathogenic potential of wild-type *P. sabiae* was tested by infecting the model organism Galleria mellonella. In contrast to B. cenocepacia H111, no significant virulence was observed (Table S5).

The fact that *P. sabiae* can reduce the soft rot disease caused by *P. carotovorum* on two varieties of potato tubers (Anabelle and Celtiane) demonstrates the potential to use this *Paraburkholderia* strain for phytopathogen biocontrol (Fig. 7, Fig. S5). *P. carotovorum* is a pathogen found on all continents and belongs to the SRE, which cause soft rot in 50% of angiosperm plant orders, including important food crops such as potato, pepper, celery, and tobacco (26, 53–58). Potato (S. tuberosum *L.*) is an affordable crop and the third most important plant in agriculture after rice and wheat (59, 60). Bacterial infections imply a significant biotic constraint, and biological control by microbial inoculants is an effective and inexpensive alternative for control of tuber soft rot compared to genetically modified potato plants, physical seed tuber treatment, and chemical seed treatment (61). In contrast to the clear T6SS-1-mediated killing of the phytopathogens *in vitro* (Fig. 1), *P. sabiae* T6SS-1 cannot entirely protect the potato tuber from *P. carotovorum* attack (Fig. 7B), as a T6SS-1 mutant still rescued around 33% of the potatoes. This suggests that additional bacterial factors also play a role in the protective effect of *P. sabiae*. Our preliminary results suggested that the second T6SS of *P. sabiae* (T6SS-3, Fig. S6) is not involved in protection against *P. carotovorum*-caused soft rot in the potato. Additionally, abiotic factors such as pH and oxygen levels can also influence the progress of soft root disease. For example, the pectate lyase activity of D. dadantii (*pelC*), which is the main reason for the plant tissue maceration and whose gene is also found in *P. carotovorum* (62), is highly pH-dependent (63). One possibility is that the presence of *P. sabiae* modulates the pH of the environment and influences the growth and pathogenicity of the phytopathogen.

To gain further insights into the genetics underlying *P. sabiae*’s protective effect against important phytopathogens, a combination of functional genomics approaches could be used in the future. While proteomics is the method of choice to identify the effectors of the key component T6SS-1, a combination of dual RNA-sequencing and metabolomics on healthy and rotten potato tubers would provide valuable information about the molecular mechanisms underlying biocontrol activities and crop responses. Finally, we are currently exploring the value of *P. sabiae* as a potential biocontrol agent for important crops affected by pathogens that are also targeted by *P. sabiae*, such as P. syringae, R. solanacearum, and *B. gladioli*.

## MATERIALS AND METHODS

### Bacterial strains, media, and cultivation.

The strains, plasmids and primers used in this study are listed in Table S1. All *Paraburkholderia* and target strains were grown in LB medium without salt at 28°C and 180 rpm (64). LB medium (65) was used for all Escherichia coli strains. The appropriate concentrations of antibiotics were added to the media as needed: chloramphenicol (Cm), 20 μg/mL for E. coli and 80 μg/mL for *P. sabiae*; and gentamicin (Gm), 20 μg/mL for Tn7 strains. The expression of the promoter fusions was observed in AB minimal medium (66) with different carbon sources (15 mM succinate, malate, or fumarate).

### Competition assay *in vitro*.

To find antagonistic interactions between strains, we first screened different strains with a drop assay on LB plates without salt. The bacteria were grown overnight in liquid LB without salt, washed twice with MgSO_4_ and normalized to an OD_600_ (optical density at 600 nm) of 1. Next, 10-μL volumes of each strain were dropped next to each other on an agar plate, creating a contact zone. The contact zone was analyzed after 24 h under a compound microscope and images were taken with a Lumenera Infinity 3-1 Digital Camera. Interbacterial competition was further tested by modified killing assays on plate, as described previously (9). In brief, bacteria were grown overnight in liquid LB without salt, washed twice with MgSO_4_ and normalized to OD_600_ = 1 for the attacker and OD_600_ = 0.1 for the target. The strains were mixed at a 10:1 ratio and 20 μL was spotted on top of cellulose nitrate filters (Cellulose Nitrate Membrane Filters, Whatman Co., cat no. 7182-002) on LB plates without salt. After 24 h of incubation at 28°C, the bacteria were recovered in 1 mL MgSO_4_, diluted (10^−1^ to 10^−6^), and plated on LB plates without salt and on selective plates (LB without salt with gentamicin for Tn7 tagged strains).

### Genome sequencing assembly and annotation.

Genomic DNA (gDNA) was extracted with the GenElute Bacterial Genomic DNA kit from Sigma-Aldrich (PCode: 1002747771). The genome was sequenced with a PacBio Sequel instrument and one SMRT Cell 1M at the Functional Genomics Center Zurich (FGCZ). Low-quality reads and reads shorter than 9,500 bp were filtered out using filtlong v0.2.0 (67); the filtered reads were then assembled using Flye v2.8.1 (68), including three polishing iterations to remove sequencing errors. Finally, an additional round of polishing was performed using Arrow (69) for reads longer than 1,000 bp. The filtered reads were mapped to the polished assembly to manually inspect the assembly for errors using IGV (Integrated Genome Viewer); the quality of the assembly was further evaluated using Qualimap v2.2.2a (70) and Sniffles v1.0.12 (71). The final assembly was annotated with Prokka, a local installation of the NCBI’s Prokaryotic Genome Annotation Pipeline (72), (PGAP 2020-09-24) and emapper v2.0.1b using the eggNOG database v5.0.2 (73). The resulting two chromosomes and two plasmids were start-aligned, i.e., the nucleotide numbering was adjusted such that position 0 of each contig represents the start of a gene: *dnaA* for chromosome 1 (following a standard approach), and the respective homologs of *P. phymatum* STM815 genes for chromosome 2 (BGD510305, paras_006859), megaplasmid1 (BGD513640 [hypE], paras_000001) and megaplasmid2 (BGD513637, paras_006467). An excel spreadsheet (Supplemental File S1) and GFF files are provided which allow researchers to compare our first local PGAP annotation (PGAP 2020-09-24, Supplemental File 2) and the NCBI annotation (2023-06-13) (Supplemental File 3), also provided as GenBank files (Supplemental Files 4 and 5) with the most recent PGAP annotation from the NCBI. Genome annotations, even those from different releases of the same annotation pipeline (here, the NCBI PGAP), are known to differ slightly (74).

### Construction of mutant strains.

An insertion mutant was constructed in the respective *tssC* genes of T6SS-1 (*paras_007259)* and T6SS-3 (*paras_007827*). For each mutant, a fragment was amplified by PCR using *P. sabiae* LMG24235 gDNA with the primer pairs TssC-1_F_EcoR1/TssC-1_R_Sal1 (402 bp) and TssC-3_F_Xho1/TssC-3_R_Xba1 (416 bp), respectively (Table S1). After digestion, the fragments were cloned into the vector pSHAFT2, resulting in the plasmids pSHAFT2::T6SS-1_IM and pSHAFT2::T6SS-3_IM, and their correct sequence was confirmed (Microsynth, Balgach, St. Gallen, Switzerland). The two constructed plasmids were transferred to *P. sabiae* LMG24235 by triparental mating. The donor (E. coli c118 λ-pir with pSHAFT2::T6SS-1_IM or pSHAFT2::T6SS-1_IM) was mixed at a 1:1:1 ratio with the helper strain (E. coli pRK2013, Table S1) and the recipient (*P. sabiae* WT) and inoculated on LB plates without salt at 28°C overnight. The transconjugants were selected with chloramphenicol (80 ng/μL) (75). The insertion of the plasmid was verified by PCR. Two promoter fusions for the T6SS-1 putative operons (*paras_007256* and paras_007257) were constructed with the vector pPROBE-NT as previously described (76). The promoter regions for paras_007256 (*tssJ*) and paras_007257 (tetratricopeptide containing protein) were amplified from *P. sabiae* gDNA with the primer pairs Pparas007256_XbaI_F and Pparas007256_HindIII_R, Pparas007257_HindIII_F and Pparas007257_XbaI_R, respectively (Table S1). After digestion, the fragments were cloned into pPROBE-NT (77). The successful cloning was again confirmed by sequencing (Microsynth) and transferred into *P. sabiae* by triparental mating.

### Expression analysis.

GFP expression of the promoters *paras_007256* (P1) and *paras_007257* (P2) was measured in 96-well plates (200 μL bacterial solution, OD_600_ = 0.05) with a Tecan Infinite M200 Pro as described previously (78). The protocol was slightly modified (28°C, biological triplicates) and measurements were taken during incubation for 48 h at 28°C. The strains were grown in complex medium (LB medium without salt) and in AB minimal media with different C4-dicarboxylate carbon sources (succinate, malate, fumarate).

### *In vivo* competition assay (potato tuber protection assay).

The ability to protect potato tubers was tested in S. tuberosum *L.* cv. Anabelle and Celtiane. The tubers were washed with deionized water, dried, and weighed. Afterwards, the tubers were surface-sterilized by washing with 70% ethanol and dried. The bacteria were grown overnight, washed twice with MgSO_4_, and normalized to OD_600_ = 1 (biocontrol strain: *P. sabiae* LMG24235) and OD_600_ = 0.1 (phytopathogenic target strain: *P. carotovorum* subsp. *carotovorum*), respectively. Next, 10 μL of biocontrol strain was injected directly into the potato. After 30 min of drying, 10 μL of the attacker strain was injected into the potato at the same position and the potato was dried for another 30 min. The potatoes were packed into bags and incubated at 28°C in the dark for 14 to 21 days. Pictures were taken of both the whole potato and the potato after being cut in half through the injection hole. The rotten part was scraped off and the healthy part of the potato was weighed. The data were normalized with the weight of the potato before injection.

### Bioinformatics and statistical analysis.

Several types of secretion systems (T1SS to T6SS) were predicted among the protein sequences of coding DNA sequences (CDS) annotated by PGAP (build 4894) in *P. sabiae*’s genome sequence using macsyfinder v2.0 (79) with the TXSScan model data set (36) and “ordered_replicon” as the database type. The genes predicted to belong to a secretion system by TXSScan or PGAP were merged into a single bed file and visualized for each replicon using Circos (v0.69-8) (80), also including VgrG genes as annotated by PGAP. Additionally, the replicon read coverage and GC skew were visualized. The conservation of genes of *P. sabiae*’s T6SS-1 gene cluster (chromosome 2:452,525 to 476,239, from gene *paras_007251* to *paras_007270*) was analyzed by searching a subset of their protein sequences against the NCBI’s Identical Protein Groups (IPG) resource using cblaster v1.3.17 (81). Default parameters were used except for requiring a subset of 10 core proteins of the baseplate (TssAEFGK), membrane complex (TssJLM), and sheath (TssBC, paras_007258, and paras_0072589) to be present in the gene cluster (the two genes making up the tail [tube and tip], as well as TssH were excluded because the tail tip can be located outside the gene cluster). A table of the best ortholog hits for each gene in the identified cluster and the whole genome per species and strain was compiled as previously described (43), and a synteny plot of the identified clusters from 8 strains of interest was generated using cblaster’s plot function. The protein sequences of the 16 genes common to all 9 strains were separately aligned using Clustal Omega v1.2.4 (82). Partitions were defined for each individual protein except for two proteins where the orthologs were identical in some species (*paras_007258* and *paras_007261*). In these two cases, the partitions were merged with the previous gene (*paras_007257* and *paras_007260*, respectively). The automatic model selection mode of RAxML v8.2.12 (83) (PROTGAMMAAUTO) was used to determine the best selection model for each partition. The alignments of all genes were concatenated using BioPython v1.79 (84) and RAxML was used to create 100 bootstrap trees with the best model for each partition in PROTGAMMA mode. The resulting bootstrap trees were concatenated, and bootstrap values were added to the best tree using RAxML with an -f b flag. The tree was visualized using FigTree. Phyre2 (http://www.sbg.bio.ic.ac.uk/phyre2/html/page.cgi?id=index) protein structure prediction (modeling mode: normal) was used to identify proteins similar to the TPR protein (85). Statistical significance in competition, expression, and biocontrol experiments was analyzed with GraphPad Prism v7.0. Analysis of variance (ANOVA) with Tukey’s multiple-comparison test was used to assess significantly different means in all experiments (*, *P* ≤ 0.05; **, *P* ≤ 0.01; ***, *P* ≤ 0.001; ****, *P* ≤ 0.0001).

### Data availability.

The complete, *de novo* assembled genome sequence of *P. sabiae* LMG24235 is available from the NCBI under BioProject ID PRJNA956509, with the accession numbers CP125295 to CP125298 for the 2 chromosomes and 2 megaplasmids.
