# Supplementary material for: Paraburkholderia sabiae Uses One Type VI Secretion System (T6SS-1) as a Powerful Weapon against Notorious Plant Pathogens
Source: Microbiol Spectr. 2023 Jul 13;11(4):e01622-23. doi: 10.1128/spectrum.01622-23 (PMC10434147; doi:10.1128/spectrum.01622-23)
Supplement: Supplemental file 6 — Tables S1 to S5 and Fig. S1 to S6. Download spectrum.01622-23-s0002.docx, DOCX file, 2.6 MB [file spectrum.01622-23-s0002.docx]

*Paraburkholderia sabiae* uses one Type VI Secretion System (T6SS-1) as a powerful weapon against notorious plant pathogens

Sebastian Hug^1^, Benjamin Heiniger^2^, Kim Bolli^1^, Sarah Paszti^1^, Leo Eberl^1^, Christian H. Ahrens^2*^ and Gabriella Pessi^1*^

^1^ Department of Plant and Microbial Biology, University of Zürich, CH-8057 Zürich, Switzerland

^2^ Agroscope, Molecular Ecology and Swiss Institute of Bioinformatics, CH-8046 Zurich, Switzerland, Switzerland

***Correspondence:**Christian H. Ahrens, [christian.ahrens@agroscope.admin.ch](mailto:christian.ahrens@agroscope.admin.ch)

Gabriella Pessi, [gabriella.pessi@botinst.uzh.ch](mailto:gabriella.pessi@botinst.uzh.ch)

Supplementary Materials

Contents

[Supplementary Table 1 3](#_Toc137737296)

[References 5](#_Toc137737297)

[Supplementary Table 2. 7](#_Toc137737298)

[Supplementary Table 3. 8](#_Toc137737299)

[Supplementary Table 4 11](#_Toc137737300)

[Supplementary Table 5. 12](#_Toc137737301)

[Supplementary Figure 1 13](#_Toc137737302)

[Supplementary Figure 2 14](#_Toc137737303)

[Supplementary Figure 3 15](#_Toc137737304)

[Supplementary Figure 4 16](#_Toc137737305)

[Supplementary Figure 5 17](#_Toc137737306)

[Supplementary Figure 6 18](#_Toc137737307)

**Supplementary Table 1**. Used strains, plasmids and oligonucleotides.

| Strain | Description | Reference |
| --- | --- | --- |
| *P. sabiae* LMG24235 |  |  |
| WT |  | (1) |
| T6SS-X_IM | LMG24235, *tssC (paras_007259)* insertion mutant; Cm^R^ | This study |
| T6SS-Y_IM | LMG24235, *tssC (paras_007827)* insertion mutant; Cm^R^ | This study |
| *E. coli* |  |  |
| c118 λ-pir | Δ*(ara-leu) araD* Δ*lac74 galE galK phoA20 thi1 rpsE rpoB argE(Am), recAI* λ *pir;* Strep^R^ | (2) |
| *P. phymatum* STM815 |  |  |
| WT-pPROBE | pPROBE-NT without promotor; Km^R^ | (3) |
| WT-pPROBE-p5978 | pPROBE-NT with promotor for *bphy_5978*; Km^R^ | (4) |
| *P. syringae* DC3000 |  |  |
| WT (Tn7) | Tagged with mini Tn7(Gm) PA1/04/04-egfp-a (GFP); Gm^R^ | (5) |
| *P. syringae* B728a |  |  |
| WT | Wild type | (6) |
| *P. syringae* 1448a |  |  |
| WT | Wild type | (7) |
| *P. carotovorum* LMG2404 |  |  |
| WT (Tn7) | Tagged with mini Tn7(Gm) PA1/04/04-egfp-a (GFP); Gm^R^ | (5) |
| *E. amylovora* LMG1893 |  |  |
| WT (Tn7) | Tagged with mini Tn7(Gm) PA1/04/04-egfp-a (GFP); Gm^R^ | This study  Putthapoom Lumjiaktase |
| *D. dadantii* DSM4610 |  |  |
| WT | Wild type | (8) |
| *P. putida* KT2440 |  |  |
| WT (Tn7) | Tagged with mini Tn7(Gm) PA1/04/04-egfp-a (GFP); Gm^R^ | This study |
| *P. aureofaciens* ATCC13985 |  |  |
| WT (Tn 7) | Tagged with mini Tn7(Gm) PA1/04/04-egfp-a (GFP); Gm^R^ | (5) |
| *P. simiae* WCS417 |  |  |
| WT (Tn7) | Tagged with mini Tn7(Gm) PA1/04/04-egfp-a (GFP); Gm^R^ | (5) |
| *R. solanacearum* DSM9544 |  |  |
| WT (Tn7) | Tagged with mini Tn7(Gm) PA1/04/04-egfp-a (GFP); Gm^R^ | (5) |
| *P. putida* A9rx29 |  |  |
| WT (Tn7) | Tagged with mini Tn7(Gm) PA1/04/04-egfp-a (GFP); Gm^R^ | (5) |
| *P. aeruginosa* PUPa3 |  |  |
| WT (Tn7) | Tagged with mini Tn7(Gm) PA1/04/04-egfp-a (GFP); Gm^R^ | This study |
| *P. entomophila* |  |  |
| WT (Tn7) | Tagged with mini Tn7(Gm) PA1/04/04-egfp-a (GFP); Gm^R^ | (5) |
| *P. putida* IsoF |  |  |
| WT (Tn7) | Tagged with mini Tn7(Gm) PA1/04/04-egfp-a (GFP); Gm^R^ | (5) |
| *P. putida* W2 |  |  |
| WT (Tn7) | Tagged with mini Tn7(Gm) PA1/04/04-egfp-a (GFP); Gm^R^ | (5) |
| *B. plantarii* LMG9035 |  |  |
| WT | Wild type | (9) |
| *B. gladioli* LMG11626 |  |  |
| WT | Wild type | (10) |
| *B. glumae* AU6208 |  |  |
| WT | Wild type | (11) |
| *B. glumae* LMG2196 |  |  |
| WT | Wild type | (12) |
| Plasmids | **Description** | **Reference** |
| pRK2013 | Helper plasmid; Km^R^ | (13) |
| pSHAFT2 | Broad-host-range suicide plasmid; Cm^R^ | (14) |
| pSHAFT2::T6SS-1_IM | pSHAFT2 carrying a 402 bp fragment for *paras_007259* insertion mutant | This study |
| pSHAFT2::T6SS-3_IM | pSHAFT2 carrying a 416 bp fragment for *paras_007827* insertion mutant | This study |
| pUC18T-mini-Tn7T-Gm-*egfp-a* | mini Tn7(Gm) PA1/04/04-egfp-a for GFP tagging; Amp^R^, Gm^R^ | (15) |
| pUC18T-mini-Tn7T-Gm-*mCherry* | mini Tn7(Gm) PA1/04/04-mCherry for mCherry tagging; Amp^R^, Gm^R^ | (15) |
| pUX-BF13 | *tnsA-E*; helper plasmid providing the Tn7 transposition functions; Amp^R^ | (16) |
| pSU11 | *lacZ* reporter plasmid; Gm^R^ | (17) |
| pPROBE-NT | Broad-hoast-range promotor-probe vector; Km^R^ | (18). |
| pPROBE-NT::paras_007256 | pPROBE with paras_007256 promotor region in front of *gfp*; Km^R^ | This study |
| pPROBE-NT::paras_007257 | pPROBE with paras_007257 promotor region in front of *gfp*; Km^R^ | This study |
| Oligonucleotide | **Sequence** | **Reference** |
| TssC-1_F_EcoRI | GCGCgaattcAGAAGAGTTCGGCACATTCG | This study |
| TssC-1_R_SalI | GCGCgtcgacGCGTTACACCACAGGTACT | This study |
| TssC-1_U_veri | ATACGGGTTCGACGATCAAG | This study |
| TssC-1_D_veri | GCGATTTCAGTCGGACACTT | This study |
| TssC-3_F_XhoI | GCGCctcgagTGTCTCGTCGCGGATTACT | This study |
| TssC-3_R_XbaI | GCGCtctagaCAGCCATACAGCTTGAACGA | This study |
| TssC-3_U_veri | TGAAGCGCTACAAGGGTCTT | This study |
| TssC-3_D_veri | GGTCGGGCACTTCATGTC | This study |
| Pparas007256_XbaI_F | GCGCtctagaTCTGTATAGACCGGTTTTCAAG | This study |
| Pparas007256_HindIII_R | GCGCaagcttCTTTGTTTCACCGCCGTGC | This study |
| Pparas007257_HindIII_F | GCGCaagcttTCTGTATAGACCGGTTTTCAAG | This study |
| Pparas007257_XbaI_R | GCGCtctagaCTTTGTTTCACCGCCGTGC | This study |
|  |  |  |

References

1. Chen W-M, Faria SM de, Chou J-H, James EK, Elliott GN, Sprent JI, Bontemps C, Young JPW, Vandamme P. 2008. *Burkholderia sabiae* sp. nov., isolated from root nodules of *Mimosa caesalpiniifolia*. Int J Syst Evol Microbiol 58:2174–2179. doi:10.1099/ijs.0.65816-0.

2. Herrero M, Lorenzo V de, Timmis KN. 1990. Transposon vectors containing non-antibiotic resistance selection markers for cloning and stable chromosomal insertion of foreign genes in gram-negative bacteria. J. Bacteriol. 172:6557–6567. doi:10.1128/jb.172.11.6557-6567.1990.

3. Liu Y, Bellich B, Hug S, Eberl L, Cescutti P, Pessi G. 2020. The exopolysaccharide cepacian plays a role in the establishment of the *Paraburkholderia phymatum - Phaseolus vulgaris* symbiosis. Front. Microbiol. 11. doi:10.3389/fmicb.2020.01600.

4. Hug S, Liu Y, Heiniger B, Bailly A, Ahrens CH, Eberl L, Pessi G. 2021. Differential expression of *Paraburkholderia phymatum t*ype VI secretion systems (T6SS) suggests a role of T6SS-b in early symbiotic interaction. Front. Plant. Sci. 12:699590. doi:10.3389/fpls.2021.699590.

5. Purtschert-Montenegro G, Cárcamo-Oyarce G, Pinto-Carbó M, Agnoli K, Bailly A, Eberl L. 2022. *Pseudomonas putida* mediates bacterial killing, biofilm invasion and biocontrol with a type IVB secretion system. Nat Microbiol 7:1547–1557. doi:10.1038/s41564-022-01209-6.

6. Feil H, Feil WS, Chain P, Larimer F, DiBartolo G, Copeland A, Lykidis A, Trong S, Nolan M, Goltsman E, Thiel J, Malfatti S, Loper JE, Lapidus A, Detter JC, Land M, Richardson PM, Kyrpides NC, Ivanova N, Lindow SE. 2005. Comparison of the complete genome sequences of *Pseudomonas syringae* pv. *syringae* B728a and pv. tomato DC3000. Proc. Natl. Acad. Sci. U.S.A. 102:11064–11069. doi:10.1073/pnas.0504930102.

7. Owen JG, Ackerley DF. 2011. Characterization of pyoverdine and achromobactin in *Pseudomonas syringae* pv. *phaseolicola* 1448a. BMC Microbiol 11:218. doi:10.1186/1471-2180-11-218.

8. Samson R, Legendre JB, Christen R, Saux MF-L, Achouak W, Gardan L. 2005. Transfer of *Pectobacterium chrysanthemi* (Burkholder et al. 1953) Brenner et al. 1973 and *Brenneria paradisiaca* to the genus *Dickeya* gen. nov. as *Dickeya chrysanthemi* comb. nov. and *Dickeya paradisiaca* comb. nov. and delineation of four novel species, *Dickeya dadantii* sp. nov., *Dickeya dianthicola* sp. nov., *Dickeya dieffenbachiae* sp. nov. and *Dickeya zeae* sp. nov. Int J Syst Evol Microbiol 55:1415–1427. doi:10.1099/ijs.0.02791-0.

9. URAKAMI T, ITO-YOSHIDA C, ARAKI H, KIJIMA T, SUZUKI K-I, KOMAGATA K. 1994. Transfer of *Pseudomonas plantarii* and *Pseudomonas glumae* to *Burkholderia* as *Burkholderia* spp. and description of *Burkholderia vandii* sp. nov. International Journal of Systematic Bacteriology 44:235–245. doi:10.1099/00207713-44-2-235.

10. Yabuuchi E, Kosako Y, Oyaizu H, Yano I, Hotta H, Hashimoto Y, Ezaki T, Arakawa M. 1992. Proposal of *Burkholderia* gen. nov. and transfer of seven species of the genus *Pseudomonas* homology group II to the new genus, with the type species *Burkholderia cepacia* (Palleroni and Holmes 1981) comb. nov. Microbiol Immunol 36:1251–1275. doi:10.1111/j.1348-0421.1992.tb02129.x.

11. Devescovi G, Bigirimana J, Degrassi G, Cabrio L, LiPuma JJ, Kim J, Hwang I, Venturi V. 2007. Involvement of a quorum-sensing-regulated lipase secreted by a clinical isolate of *Burkholderia glumae* in severe disease symptoms in rice. Appl. Environ. Microbiol. 73:4950–4958. doi:10.1128/aem.00105-07.

12. Fory PA, Triplett L, Ballen C, Abello JF, Duitama J, Aricapa MG, Prado GA, Correa F, Hamilton J, Leach JE, Tohme J, Mosquera GM. 2014. Comparative analysis of two emerging rice seed bacterial pathogens. Phytopathology 104:436–444. doi:10.1094/PHYTO-07-13-0186-R.

13. Figurski DH, Helinski DR. 1979. Replication of an origin-containing derivative of plasmid RK2 dependent on a plasmid function provided in trans. Proc. Natl. Acad. Sci. U.S.A. 76:1648–1652. doi:10.1073/pnas.76.4.1648.

14. Shastri S, Spiewak HL, Sofoluwe A, Eidsvaag VA, Asghar AH, Pereira T, Bull EH, Butt AT, Thomas MS. 2017. An efficient system for the generation of marked genetic mutants in members of the genus *Burkholderia*. Plasmid 89:49–56. doi:10.1016/j.plasmid.2016.11.002.

15. Choi K-H, Schweizer HP. 2006. mini-Tn7 insertion in bacteria with single attTn7 sites: example *Pseudomonas aeruginosa*. Nat Protoc 1:153–161. doi:10.1038/nprot.2006.24.

16. Koch B, Jensen LE, Nybroe O. 2001. A panel of Tn7-based vectors for insertion of the gfp marker gene or for delivery of cloned DNA into Gram-negative bacteria at a neutral chromosomal site. Journal of Microbiological Methods 45:187–195. doi:10.1016/S0167-7012(01)00246-9.

17. O'Grady EP, Viteri DF, Malott RJ, Sokol PA. 2009. Reciprocal regulation by the CepIR and CciIR quorum sensing systems in *Burkholderia cenocepacia*. BMC Genomics 10:441. doi:10.1186/1471-2164-10-441.

18. Miller WG, Leveau JHJ, Lindow SE. 2000. Improved gfp and inaZ Broad-Host-Range Promoter-Probe Vectors. Mol. Plant. Microbe. Interact. 13:1243–1250. doi:10.1094/MPMI.2000.13.11.1243.

19. Lindström K, Mousavi SA. 2020. Effectiveness of nitrogen fixation in rhizobia. Microb Biotechnol 13:1314–1335. doi:10.1111/1751-7915.13517.

**Supplementary Table 2.** The genome was analysed for *nod, nif* and *fix* genes, which play a key role in plant root nodule formation and nitrogen fixation (19).

| Nod genes | Gene identifier |
| --- | --- |
| *nodA* | *paras_000453* |
| *nodB* | *paras_000458* |
| *nodC* | *paras_000457* |
| *nodH* | *paras_000454* |
| *nodI* | *paras_000456* |
| *nodJ* | *paras_000455* |
| *nodS* | *paras_000452* |
| *nodU* | *paras_000451* |
| Nif genes | **Gene identifier** |
| *nifA* | *paras_000464* |
| *nifB* | *paras_000478* |
| *nifD* | *paras_000505* |
| *nifE* | *paras_000465* |
| *nifH* | *paras_000504* |
| *nifH* | *paras_000012* |
| *nifK* | *paras_000506* |
| *nifN* | *paras_000466* |
| *nifQ* | *paras_000470* |
| *nifT* | *paras_000482* |
| *nifT* | *paras_000524* |
| *nifV* | *paras_000477* |
| *nifW* | *paras_000476* |
| *nifX* | *paras_000467* |
| *nifZ* | *paras_000481* |
| *nifZ* | *paras_000523* |
| Fix genes | **Gene identifier** |
| *fixA* | *paras_000475* |
| *fixB* | *paras_000474* |

**Supplementary Table 3.** Several secretion systems (T1SS-T6SS) were predicted in the genome using TXSScan. Further tssI copies were annotated by PGAP and are marked with an asterisk.

| Secretion system | Gene | Replicon | Gene identifier |
| --- | --- | --- | --- |
| T1SS | *abc* | Chromosome 1 | *paras_003449* |
| T1SS | *mfp* | Chromosome 1 | *paras_003451* |
| T1SS | *Omf* | Chromosome 1 | *paras_003453* |
| T2SS | *gspE* | Chromosome 1 | *paras_006434* |
| T2SS | *gspF* | Chromosome 1 | *paras_006435* |
| T2SS | *gspC* | Chromosome 1 | *paras_006436* |
| T2SS | *gspG* | Chromosome 1 | *paras_006437* |
| T2SS | *gspH* | Chromosome 1 | *paras_006438* |
| T2SS | *gspI* | Chromosome 1 | *paras_006439* |
| T2SS | *gspJ* | Chromosome 1 | *paras_006440* |
| T2SS | *gspK* | Chromosome 1 | *paras_006441* |
| T2SS | *gspL* | Chromosome 1 | *paras_006442* |
| T2SS | *gspM* | Chromosome 1 | *paras_006443* |
| T2SS | *gspN* | Chromosome 1 | *paras_006444* |
| T3SS-A | *sctC* | Chromosome 2 | *paras_008267* |
| T3SS-A | *sctT* | Chromosome 2 | *paras_008269* |
| T3SS-A | *sctN* | Chromosome 2 | *paras_008271* |
| T3SS-A | *sctJ* | Chromosome 2 | *paras_008274* |
| T3SS-A | *sctU* | Chromosome 2 | *paras_008277* |
| T3SS-A | *sctV* | Chromosome 2 | *paras_008278* |
| T3SS-A | *sctQ* | Chromosome 2 | *paras_008280* |
| T3SS-A | *sctR* | Chromosome 2 | *paras_008281* |
| T3SS-A | *sctS* | Chromosome 2 | *paras_008282* |
| T3SS-B | *sctQ* | Chromosome 2 | *paras_008521* |
| T3SS-B | *sctR* | Chromosome 2 | *paras_008522* |
| T3SS-B | *sctU* | Chromosome 2 | *paras_008525* |
| T3SS-B | *sctT* | Chromosome 2 | *paras_008526* |
| T3SS-B | *sctN* | Chromosome 2 | *paras_008528* |
| T3SS-B | *sctJ* | Chromosome 2 | *paras_008531* |
| T3SS-B | *sctC* | Chromosome 2 | *paras_008535* |
| T3SS-B | *sctS* | Chromosome 2 | *paras_008536* |
| T3SS-B | *sctV* | Chromosome 2 | *paras_008537* |
| T4SS-1 | *virB9* | Megaplasmid 1 | *paras_000223* |
| T4SS-1 | *virB8* | Megaplasmid 1 | *paras_000224* |
| T4SS-1 | *virB6* | Megaplasmid 1 | *paras_000225* |
| T4SS-1 | *virB5* | Megaplasmid 1 | *paras_000227* |
| T4SS-1 | *virb4* | Megaplasmid 1 | *paras_000228* |
| T4SS-1 | *virB3* | Megaplasmid 1 | *paras_000229* |
| T4SS-1 | *virB1* | Megaplasmid 1 | *paras_000231* |
| T4SS-2 | *virB1* | Chromosome 1 | *paras_001893* |
| T4SS-2 | *virB2* | Chromosome 1 | *paras_001894* |
| T4SS-2 | *virB3* | Chromosome 1 | *paras_001895* |
| T4SS-2 | *virB4* | Chromosome 1 | *paras_001896* |
| T4SS-2 | *virB6* | Chromosome 1 | *paras_001897* |
| T4SS-2 | *virB5* | Chromosome 1 | *paras_001898* |
| T4SS-2 | *virB8* | Chromosome 1 | *paras_001899* |
| T4SS-2 | *virB9* | Chromosome 1 | *paras_001900* |
| T4SS | *traD* | Plasmid 2 | *paras_006678* |
| T4SS | *traI* | Plasmid 2 | *paras_006679* |
| T4SS | *virB4* | Plasmid 2 | *paras_006682* |
| T5bSS | translocator | Chromosome 1 | *paras_002828* |
| T5bSS | translocator | Chromosome 1 | *paras_003115* |
| T5bSS | translocator | Chromosome 2 | *paras_007897* |
| T5bSS | translocator | Chromosome 2 | *paras_008002* |
| T5cSS | PF03895 | Chromosome 1 | *paras_001640* |
| T5cSS | PF03895 | Chromosome 1 | *paras_001680* |
| T5cSS | PF03895 | Chromosome 1 | *paras_002300* |
| T5cSS | PF03895 | Chromosome 1 | *paras_005918* |
| T6SS-1 | *tssD* | Chromosome 2 | *paras_007251* |
| T6SS-1 | *tssL* | Chromosome 2 | *paras_007254* |
| T6SS-1 | *tssK* | Chromosome 2 | *paras_007255* |
| T6SS-1 | *tssJ* | Chromosome 2 | *paras_007256* |
| T6SS-1 | *tssB* | Chromosome 2 | *paras_007258* |
| T6SS-1 | *tssC* | Chromosome 2 | *paras_007259* |
| T6SS-1 | *tssD* | Chromosome 2 | *paras_007260* |
| T6SS-1 | *tssE* | Chromosome 2 | *paras_007261* |
| T6SS-1 | *tssF* | Chromosome 2 | *paras_007262* |
| T6SS-1 | *tssG* | Chromosome 2 | *paras_007263* |
| T6SS-1 | *tssH* | Chromosome 2 | *paras_007264* |
| T6SS-1 | *tssL* | Chromosome 2 | *paras_007266* |
| T6SS-1 | *tssM* | Chromosome 2 | *paras_007268* |
| T6SS-1 | *tssF* | Chromosome 2 | *paras_008380* |
| T6SS-1 | *tssI* | Chromosome 2 | *paras_008381* |
| T6SS-1 | *evpJ* | Chromosome 2 | *paras_008385* |
| T6SS-3 | *tssM* | Chromosome 2 | *paras_007820* |
| T6SS-3 | *tssL* | Chromosome 2 | *paras_007821* |
| T6SS-3 | *tssK* | Chromosome 2 | *paras_007822* |
| T6SS-3 | *tssJ* | Chromosome 2 | *paras_007823* |
| T6SS-3 | *tssD* | Chromosome 2 | *paras_007824* |
| T6SS-3 | *tssB* | Chromosome 2 | *paras_007828* |
| T6SS-3 | *tssH* | Chromosome 2 | *paras_007829* |
| T6SS-3 | *tssE* | Chromosome 2 | *paras_007832* |
| T6SS-3 | *tssF* | Chromosome 2 | *paras_007833* |
| T6SS-3 | *tssG* | Chromosome 2 | *paras_007834* |
| T6SS-3 | *tssI* | Chromosome 2 | *paras_007839* |
| T6SS-3 | *tssI* | Chromosome 2 | *paras_007855* |
| T6SS-3 | *evpJ* | Chromosome 2 | *paras_007858* |
| - | *tssI** | Megaplasmid 1 | *paras_000364* |
| - | *tssI** | Chromosome 1 | *paras_002442* |
| - | *tssI** | Chromosome 1 | *paras_002998* |
| - | *tssI** | Chromosome 1 | *paras_005637* |
| - | *tssI** | Chromosome 2 | *paras_008754* |

**Supplementary Table 4**. *P. sabiae* killing range. Strains with * are labelled with Tn7.

| Strain | CFU reduction |
| --- | --- |
| *Burkholderiaceae* |  |
| *Parabutkholderia tuberum* LMG21444 | 10^7^ |
| *Paraburkholderia phymatum* STM815 WT-pPROBE | 10^4^ |
| *Paraburkholderia mimosarum* LMG23256 *(*pBAH8) | 10^4^ |
| *Ralstonia solanacearum* DSM9544* | 10^3^ |
| *Paraburkholderia phytofirmans* PsJN | 10^3^ |
| *Paraburkholderia caribensis* LMG18531 (pBAH8) | 10^2^ |
| *Burkholderia plantarii* LMG9035 | 10^2^ |
| *Burkholderia gladioli* LMG11626 | 10^2^ |
| *Burkholderia tropica* LMG22274 (pBAH8) | 10^1^ |
| *Paraburkholderia nodosa* LMG23741 (pBAH8) | 10^1^ |
| *Burkholderia glumae* LMG2196 | - |
| *Burkholderia glumae* AU6208 | - |
| *Pseudomonas aeruginosa* PUPa3* | - |
| *Burkholderia cenocepacia* H111 | - |
| *Pseudomonadaceae* |  |
| *Pseudomonas syringae* DC3000* | 10^9^ |
| *Pseudomonas putida* KT2440* | 10^3^ |
| *Pseudomonas syringae* 1448a | 10^3^  10^3^  **2** |
| *Pseudomonas syringae* B728a | 10^2^  **2** |
| *Pseudomonas simiae* WCS417* | 10^2^ |
| *Pseudomonas aureofaciens* ATCC13985* | 10^2^ |
| *Pseudomonas putida* A9rx29* | 10^1^ |
| *Pseudomonas putida* W2*** | - |
| *Pseudomonas aeruginosa* PUPa3*** | - |
| *Pseudomonas putida* IsoF*** | - |
| *Pseudomonas entomophila** | - |
| *Enterobacteriaceae* |  |
| *Pectobacterium carotovorum*  LMG2404*** | 10^4^ |
| *Erwinia amylovora* LMG1893*** | 10^3^ |
| *Dickeya dadantii* DSM4610 | 10^1^ |
| *Xanthomonas campestris* | 10^1^ |
| *Pectobacterium atrosepticum* DSM9455 | - |

**Supplementary Table 5.** Various phenotypical characterization assays showed no significant differences for *P. sabiae* wt and T6SS-1_IM mutant.

| Phenotypical characterization | Description |
| --- | --- |
| Biofilm formation | Cristal violet (CV) assay |
| Motility | LB without salt, 0.2% agar |
| Salt stress | LB without salt, salt gradient plate |
| Symbiotic properties (*Mimosa pudica*) | 28 days post inoculation |
| Number of nodules | - |
| Total weight of nodules | - |
| Weight per nodule | - |
| Nitrogen fixation | Acetylen reduction assay(ARA) assay |
| Pathogenicity assay | *Galleria mellonella* |


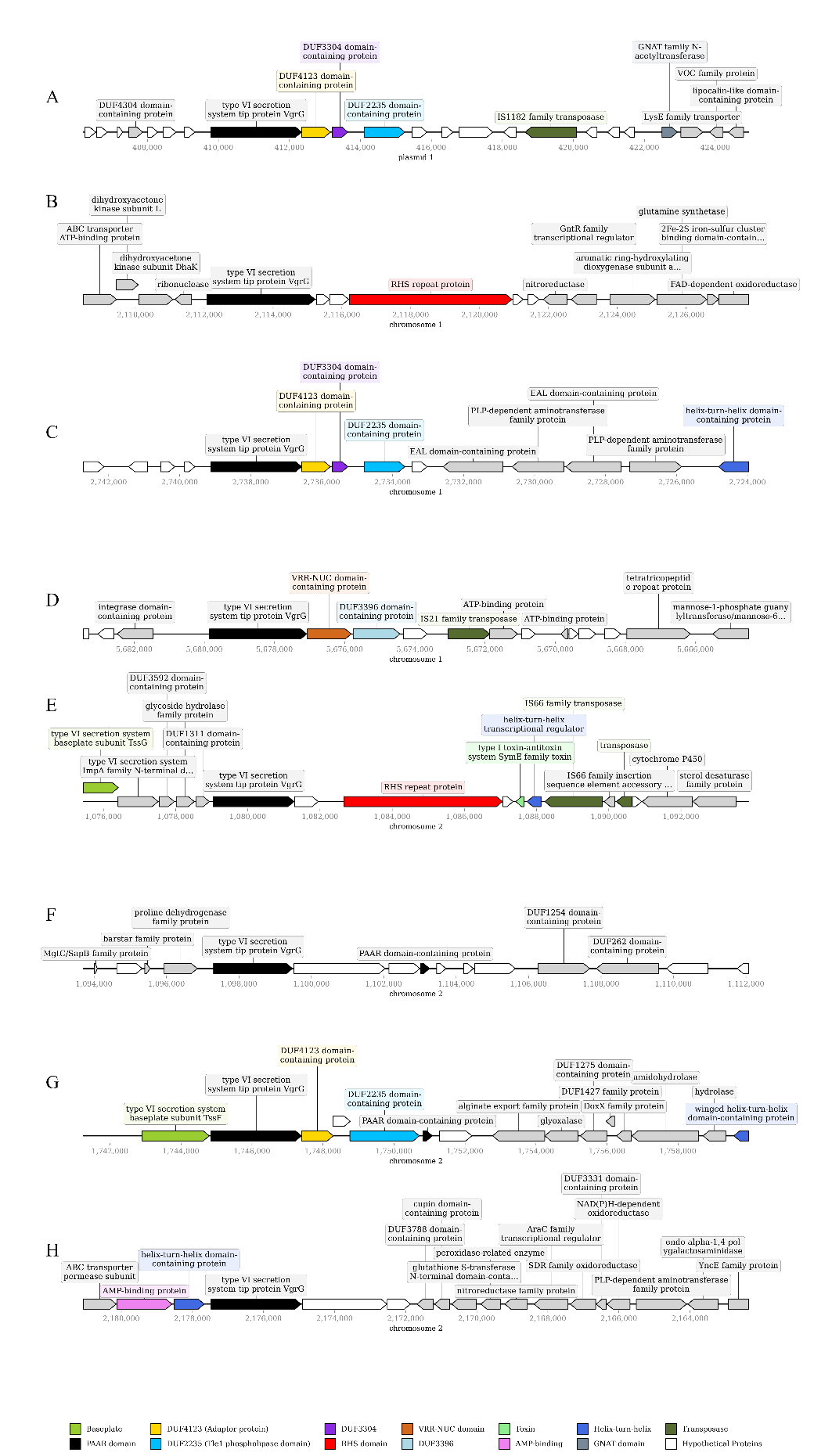


# Supplementary Figure 1. Physical map of all *tssI* loci of *P. sabiae: (*A) *paras_000364,* (B) *paras_002442,* (C) *paras_002998,* (D) *paras_005637,* (E) *paras_007839,* (F) *paras_007855,* (G) *paras_008381,* (H) *paras_008754*.


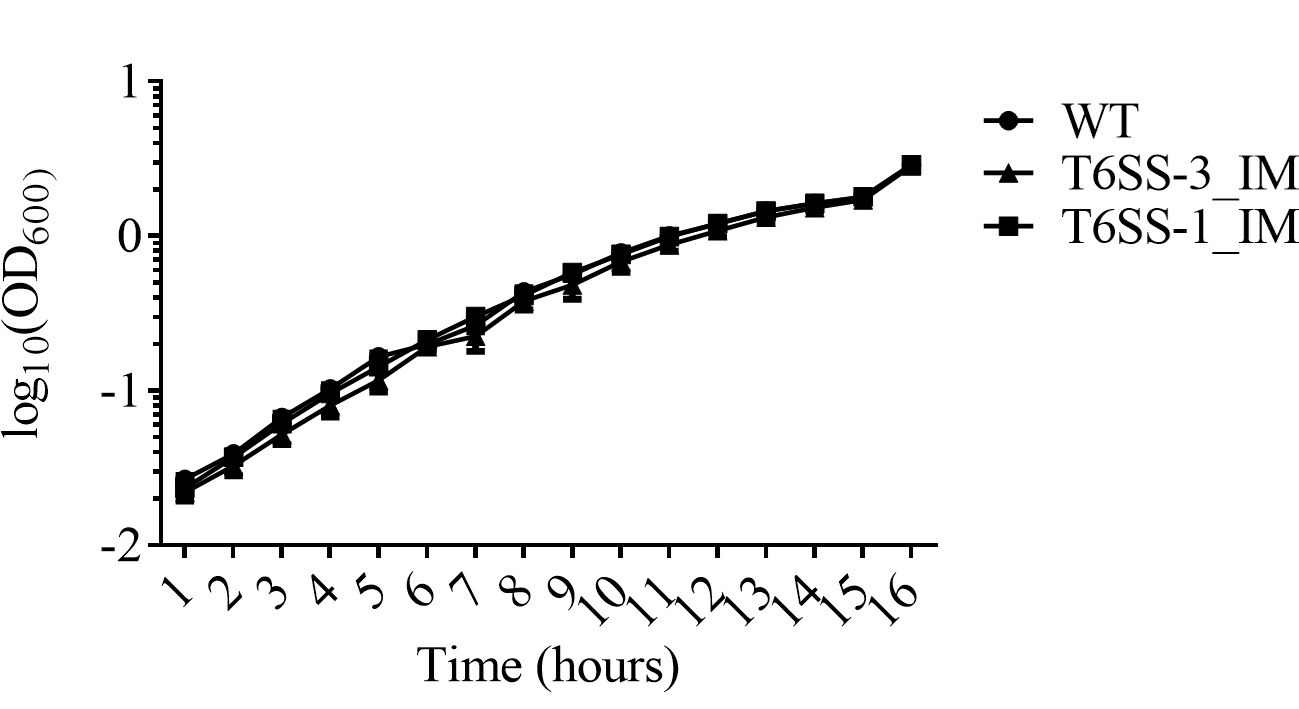


# Supplementary Figure 2. The growth of three strains (*P. sabiae* WT, T6SS-1_IM, T6SS-3_IM) was monitored in LB without salt over 16 hours. No differences were observed between the wild-type and the mutant strains.


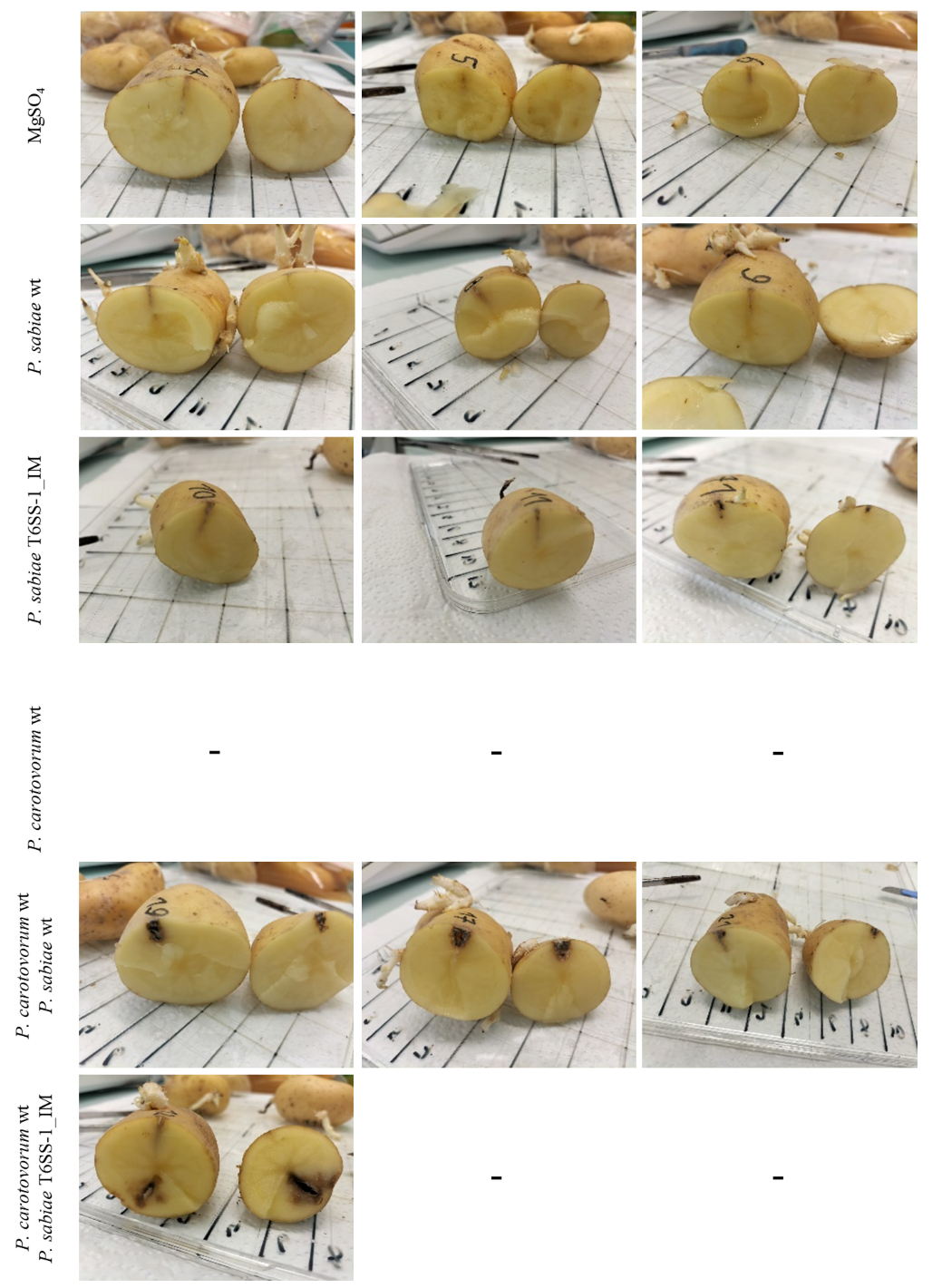


# Supplementary Figure 3. Pictures of the tubers (Celtiane, one per biological replicate) cut in half through the injection hole. Pictures of fully liquified tubers were not taken (-).


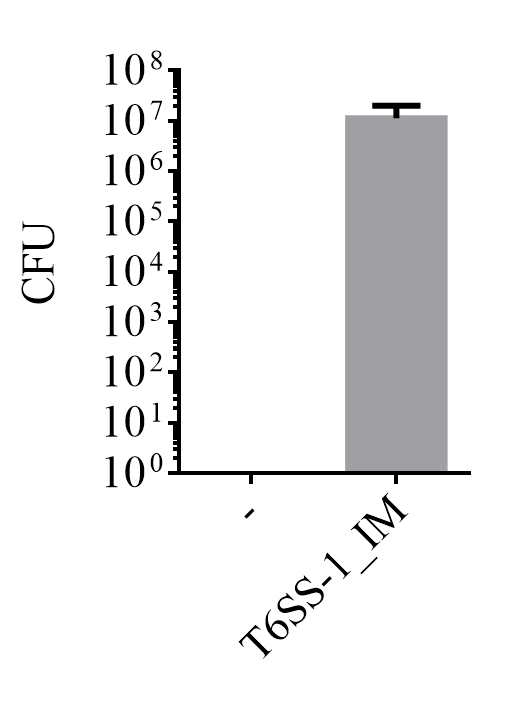


# Supplementary Figure 4. Recovered *P. sabiae* T6SS-1_IM from a potato infection site (Celtiane) after 2 weeks on LB without salt selected with chloramphenicol. An uninfected potato was used as a negative control.

**
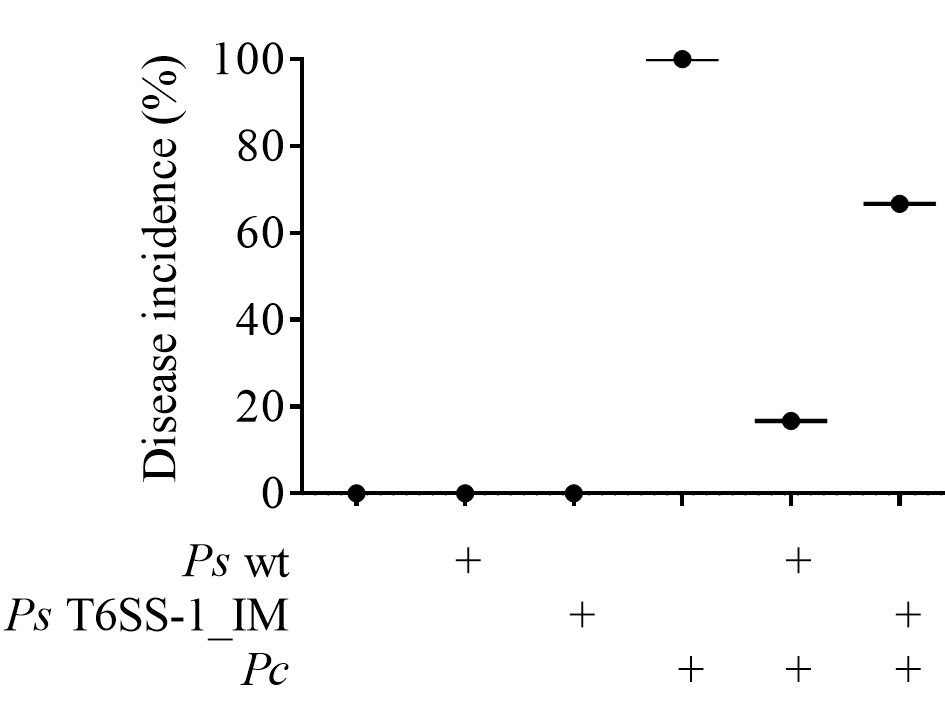
**

# Supplementary Figure 5. *P. sabiae* protects potato tuber against the phytopathogen *P. carotovorum*. Potato tubers (Anabelle) were inoculated and incubated at 28°C for 2 weeks. The co-inoculation was executed at the same spot. First, the biocontrol strain *P. sabiae* wild type (*Ps* wt) or *P. sabiae* T6SS-1 mutant (*Ps* T6SS-1_IM) was injected with a pipette tip to inflict mechanical damage to the potato. After 30 minutes, the soft rot bacteria *P. carotovorum* (*Pc*) was added to the mechanically damaged spot. The disease incidence of the potato tubers from the Anabelle biovar. (controls: 3 potatoes, competition: 6 potatoes). The percentage is taken from the total amount of tubers.


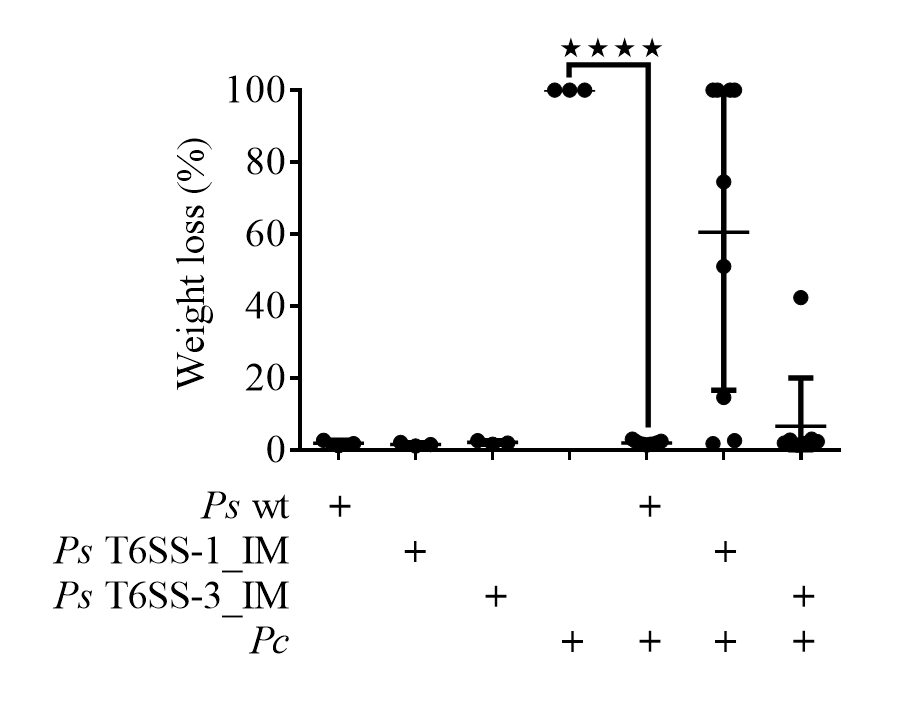


# Supplementary Figure 6. Potato tubers (Celtiane) were inoculated and incubated at 28°C for 2 weeks. The co-inoculation was executed at the same spot. First, the biocontrol strain *P. sabiae* wild type (*Ps* wt), *P. sabiae* T6SS-1 mutant (*Ps* T6SS-1_IM) or T6SS-3 mutant (*Ps* T6SS-3_IM) was injected with a pipette tip to inflict mechanical damage to the potato. After 30 minutes, the soft rot bacteria *P. carotovorum* (*Pc*) was added to the mechanically damaged spot. The rotten tissue of the potato tubers (Celtiane) was removed and the healthy tissue was weighed. A one-way ANOVA with Tukey’s multiple comparisons test was used to analyze biological triplicates (controls: 1 potato/replicate, competition: 3 potatoes/replicate).
